# A Day Off

**Published:** 1884-10

**Authors:** Geo. E. Blackham


### A DAY OFF.

GEO. E. BLACKHAM, M. D.

Come, my friend! Don't you hear the strong shrill voice of my calliope; the wheelman's bugle call! Come, up from your slumbers! Don the laced-up woolen shirt, the knee breeches, long stockings and low shoes that we wheelmen alone in these degenerate days are privileged to wear! Come, the sun is up and the autumn landscape glows with the glorious tints of ripened fruit and changing leaf. The clear, pure air is cool and bracing and the roads are smooth and almost dustless. Shake off dull sloth and sordid business cares. The Arabian fable is an American fact and the enchanted horse awaits his rider. Ere we start let us glance at our steeds as they stand together, their slender spokes gleaming like dewy cobwebs in the morning sun, the silvery gleam of nickled head and cranks showing in rich contrast with the soft bright black of back bone and forks. See to it that bearings are well oiled and saddles firmly clamped and then vault into the saddle and away.

The city fades behind us in the distance and already we are in the country. How fast, did you say? Oh, about twelve miles an hour; we are not in a hurry this morning and so we just glide along, for the fun of it. Was ever motion more noiseless and swift? Was ever such speed attained with so little exertion? We seem to move almost without effort, the slender rubber tires touch the ground at but two points and so light is the touch and so slender the delicate frame work that supports us that we seem rather to be flying than riding.

Here is the farm house that I spoke of and here we halt for a breakfast. It is a land flowing with milk and honey, and we get our share—and an invitation to "go over into the vineyard and help yourselves," and we go. Here the rich puple of the Hartfords and Concords form a dark background for the rich, green leaves; there the golden brown bunches of the Catawbas and Dianas hang in tempting profusion within easy reach. We eat till we are satisfied and then go with our host to look at his cattle—pure Alderneys in this lot, tame, gentle and beautiful with their large soft black eyes and delicate heads looking more like some kind of antelope than cattle. They are not great milkers, but what they give is nearly like cream. Yonder is a herd of different breed, great, quiet, almost stolid looking creatures—but true aristocrats every one—each with a pedigree reaching back through many generations and across the ocean to Holstein. "There's a heifer that gives her twenty-five quarts a day," says our host. "What do you think of that for a youngster?"

But enough of this. The road stretches out invitingly before us and we have twenty miles to go before noon if we would keep our appointment for dinner, and so, with "Many thanks; good-bye," from us, and "Good-bye; come again," from our hospitable host and hostess we mount again and are off.

Now the road gets a little hilly and we slacken our pace a bit on the up grade. Here we are at the summit. Let us pause and take in the view. On our left the blue-green surface of the lake fades insensibly into the bluer blue of the sky till but for the white sails here and there we could not tell lake from sky. On our right the hills rise in billowy curves, their sides adorned with the varied beauties of field and forest—here a maple flings its scarlet banners to the breeze, and there a mass of evergreens proclaims its sturdy defiance to the artist autumn. Here is a little old farmhouse, low and rambling, but glorious with the deep red paint that was so dear to the hearts of the early settlers. Here is an orchard with its old knarled trees embossed with golden fruit. Here a mass of golden rod, and there a straggling growth of blue asters along the roadside, and there by the side of a little stream a group of tall bur marigolds hold their gaudy golden heads high above more modest plants.

To saddle once more. Half a mile further and we reach the beginning of a long stretch of down grade. The road is smooth and clear and now for a glorious coast, oh here we go, legs over handles and away. Did you ever experience anything like that before? Not since you were a boy and went coasting on the old bob sled with your best girl. But even that memory is eclipsed. No frozen fingers or glowing noses to-day, but a swift, silent rush through the fragrant air that is just cool enough to make exercise agreeable, and just warm enough to make the breeze of your motion delightful. How fast? Oh, I don't know; twenty-five miles an hour, maybe—maybe more—may be less—but fast enough to make your heartthrob and your eyes shine and to make all other modes of mundane movement seem stale, flat and unprofitable by comparison. The bottom of the hill is reached with a rush and the impetus carries us far along the level ere we are obliged to take our legs down from "over the handles" and apply our feet to the pedals again. Slack up a bit now, there comes a vehicle towards us. What is it? Some scarey horse with a still more scarey driver, perhaps, and though we know our rights and knowing, dare maintain, yet we must be considerate and courteous to those who either from choice or misfortune still cling to the antiquated equine modes of locomotion. But no; there is no horse, no driver; but, by the beard of the Prophet—it's the doctor and his wife come out to meet us on their new sociable tricycle. See how well the lady does her share and how cheek and eye glow with health, the product of exercise, sunshine and fresh air. "We thought we'd meet you before you reached our house. A lot of the young folks have a picnic in the grove a couple of miles from here, and you're invited." The sociable comes about and leads the way, and in a few minutes we reach the grove, just in time for dinner. Introductions follow, and we sit down to an *al fresco* meal tempting enough in itself but doubly so to us with our appetites sharpened by our ride.

The meal dispatched, the company breaks up into little squads to wander off into the woods, botanizing, or what not, till the sun begins to sink into the west, when a few blasts from the doctor's calliope summons us to supper, after which the band wagon and sundry buggies etc., are brought into requisition to take the picnicers home, and we follow on our wheels with the doctor and his wife in their "sociable." An hour or so is pleasantly spent at the doctor's house till the full moon is high enough to make the night almost like day, and then we re-mount our wheels and start for home.

How changed the scene from the morning. Now all is quiet, save the occasional cry of some night bird—the moonlight, bright as it is, does not reveal the details of the landscape as the daylight did, but casts a sort of enchantment over it, softening and beautifying everything. The air grows chill and the dew falls heavily and we are fain to quicken our pace to keep ourselves warm.

Swift and silent we fly onward and ere we realize it the lights of the city gleam before us. A few moments more and here we are at home. Our steeds need neither feeding nor grooming; so we stable them in their own little shed and go in to narrate the adventures of the day to the home circle, and presently to bed to sleep the sleep of the just; tired, but not over tired by our sixty miles in the saddle since sunrise.

"Good night, old boy." "Good night. Glorious day, wasn't it?" "Glorious indeed." "Let's take another day off pretty soon." "Yes, if we can get it. Good night."

